# Automatic Detection and Association Analysis of Multiple Surface Defects on Shield Subway Tunnels

**DOI:** 10.3390/s23167106

**Published:** 2023-08-11

**Authors:** Ziren Yin, Zhanzhan Lei, Ao Zheng, Jiasong Zhu, Xiao-Zhou Liu

**Affiliations:** 1 College of Urban Transportation and Logistics, Shenzhen Technology University, Shenzhen 515118, China; 2210414014@email.szu.edu.cn; 2 College of Civil and Transportation Engineering, Shenzhen University, Shenzhen 515118, China; 2070474147@email.szu.edu.cn (Z.L.); 2060325014@email.szu.edu.cn (A.Z.); zhujiasong@gmail.com (J.Z.); 3 Institute of Urban Smart Transportation & Safety Maintenance, Shenzhen 515118, China; 4 Guangdong Provincial Key Laboratory of Urban Informatics, Shenzhen 515118, China

**Keywords:** shield subway tunnel, surface defects, 3D laser scanning, defect association analysis

## Abstract

The surface defects on a shield subway tunnel can significantly affect the serviceability of the tunnel structure and may compromise operation safety. To effectively detect multiple surface defects, this study uses a tunnel inspection trolley (TIT) based on the mobile laser scanning technique. By conducting an inspection of the shield tunnel on a metro line section, various surface defects are identified with the TIT, including water leakage defects, dislocation, spalling, cross-section deformation, etc. To explore the root causes of the surface defects, association rules between different defects are calculated using an improved Apriori algorithm. The results show that: (i) there are significant differences in different association rules for various surface defects on the shield tunnel; (ii) the average confidence of the association rule “dislocation & spalling → water leakage” is as high as 57.78%, indicating that most of the water leakage defects are caused by dislocation and spalling of the shield tunnel in the sections being inspected; (iii) the weakest rule appears at “water leakage → spalling”, with an average confidence of 13%. The association analysis can be used for predicting the critical defects influencing structural reliability and operation safety, such as water leakage, and optimizing the construction and maintenance work for a shield subway tunnel.

## 1. Introduction

The last few decades have seen substantial growth in the construction of urban rail transit, particularly in the southeast coastal area of China. Most of the newly built lines are underground lines with shield tunnel structures. These shield tunnels are prone to various types of defects on their surfaces due to geological, climatic, construction, and operational factors. Such defects encompass water leakage, dislocation, spalling, cross-section deformation, etc., which can pose a significant impact on the tunnel structure and may compromise the operation safety of the subway. Therefore, it is imperative to regularly inspect a subway tunnel during the construction and operation periods to detect potential defects at an early stage so that the proper measures can be used to avoid further accidents. With the rapid development of subway construction, higher requirements are put forward for the efficiency and accuracy of tunnel inspection. However, traditional manual inspection is laborious, time-consuming, and lacks informatization. To address this gap in detection techniques, extensive research has been undertaken to develop automatic detection systems. Driven by the different requirements of surface defect detection, a range of automatic detection methods have been proposed. The key detection methods include the vision-based detection method [1,2,3], laser scanning-based detection method [4,5], thermal imaging-based detection method [6], etc.

The vision-based measurement method calculates the geometric parameters of the measured object in three-dimensional space using captured images. For instance, high-resolution image information of a tunnel surface can be rapidly obtained using multiple area-scan CCD cameras, and damage can be identified and quantified using intelligent analysis methods [7]. Based on image acquisition, several acquisition systems have been developed to efficiently and accurately adapt to the actual condition of subway tunnels, such as the MTSIS system proposed by Li et al. [8] and the MTI-100 system developed by Huang et al. [9]. In terms of image data processing, deep learning techniques are frequently applied to identify surface defects [10], including full convolutional neural networks (FCNs) [11], mask R-CNN networks (R-CNN) [12,13], YOLO networks [14], etc.

However, in order to address the low-light condition in subway tunnels, lighting devices are needed, which necessitates additional preparation. The laser scanning-based method offers a solution by quickly obtaining three-dimensional coordinates and reflection intensity of numerous measuring points on the lining surface using laser ranging without the need for a light source. Various studies have been conducted to detect different types of surface defects in tunnels. For instance, Zhou et al. [15] propose an integrated approach based on laser intensity and depth features for the automated detection and quantification of concrete spalling. Xu et al. [16] extract water leakage regions in an underground tunnel using corrected intensity data and a 3D point cloud of a terrestrial laser scanning sensor. Sun et al. [17] determine the overall and local deformation of a tunnel using denoising and 360-degree deformation analysis of the point cloud data and compare the method with data collected for the total station, proving the accuracy of their method.

The thermal imaging-based method operates by converting the temperature distribution of the tunnel lining surface into a visual image using an infrared thermal imager. This technique can reveal water leakage areas and cavities in shield tunnels. For instance, Fahmy et al. [18] successfully identify a leakage area by analyzing a temperature gradient, while Yu et al. [19] propose a novel method for identifying and extracting tunnel lining cracks from infrared images. Konishi et al. [20] verify the feasibility of using thermal imaging technology to detect cavities behind the lining using a series of laboratory and field tests. However, due to the thermal imager’s sensitivity to temperature changes and the significant influence of auxiliary trackside facilities in subway tunnels on detection accuracy, this method is rarely used in practical detection projects.

In addition to defect detection, in recent years, some studies have focused on the prediction of infrastructure conditions inside subway tunnels. For instance, Sresakoolchai et al. develop deep machine learning models using three-dimensional recursive neural network-based co-simulation models to predict the next year’s track geometry parameters [21]. Liu et al. establish a random forest seepage risk prediction model, which exhibits higher accuracy compared to traditional machine learning methods such as support vector machines and artificial neural networks [22].

This study uses three-dimensional laser scanning technology to detect and identify diverse surface defects in shield tunnels. This technology proves effective in acquiring a substantial amount of highly compatible data, even in poorly lit tunnel environments. It provides three-dimensional coordinate information and reflection intensity data for measuring points. By analyzing the detection results of multiple defects, the interrelationship among them can be thoroughly examined, thereby offering insights into the underlying causes of critical defects. It is noteworthy that the existing research primarily concentrates on specific defect identification methods, with limited attention given to the correlation between different defects. To address this gap, the present paper uses the Apriori algorithm to analyze the correlation between distinct surface defects. Nine sets of association rules are established, and their respective confidence values are calculated, thus revealing the relationships among various defects.

The remainder of this paper is organized as follows. The laser scanning-based surface defect detection system for shield tunnels as well as the data processing method for identifying multiple defects is presented in Section 2. Section 3 presents the results of the association analysis for different surface defects based on the Apriori algorithm. Finally, Section 4 provides concluding remarks.

## 2. Data Acquisition and Processing

### 2.1. Types of Surface Defects on Shield Tunnels

Based on field investigations conducted on subway lines, the main surface defects can be categorized into three groups: water leakage, structural damage (including lining cracks and spalling), and structural deformation (including tunnel settlement, section convergence, and dislocation). This paper primarily focuses on the study of water leakage, dislocation, spalling, and cross-section deformation.

Water leakage is a prevalent defect in shield subway tunnels, characterized by the infiltration of water into the tunnel material due to structural defects or geological and climatic factors. Segment dislocation refers to the relative displacement of adjacent rings in the plane perpendicular to the tunnel axis or the relative displacement of adjacent segments in the radial direction. Spalling, another common tunnel defect, is primarily caused by long-term material deterioration during operation and non-compliance with concrete quality standards. Cross-section deformation arises from changes in external loads or soil erosion, which can alter and redistribute the soil stress field, ultimately impacting the stability of the soil layer surrounding the tunnel and resulting in cross-section deformation.

### 2.2. Testing Technology and Equipment

As aforementioned, compared with the vision-based detection method, which requires high lighting conditions, the laser scanning-based technology becomes more favorable under the test condition in subway tunnels. The laser scanning system can accurately detect the deformation in a subway tunnel and acquire point cloud data. The efficiency of data acquisition is high, but due to the huge amount of point cloud data, a proper data processing algorithm is needed.

It should be noted that there is a narrow time window to access a subway for a tunnel inspection, i.e., 1:00–4:00 a.m. Under such a situation, the three-dimensional laser scanning technology, characterized by fast data acquisition speed and high accuracy and needing less manpower, is used to form a tunnel inspection trolley (TIT). The system operates on the principle of using laser ranging to measure the distance between the scanner and the target point based on the round-trip time of the laser, as well as the laser’s speed and angle. The laser emitter rapidly measures the scene information inside the tunnel using high-speed rotation and stores the information in the point cloud. The point cloud data include both the coordinate information inside the tunnel and the intensity of the point cloud. In the defective area on the tunnel surface, the intensity information of the point cloud will be significantly lower than that of the surrounding area. Therefore, intensity serves as a feature to identify the defective area, which can be further located by combining the coordinate information in the point cloud.

**Figure 1:**
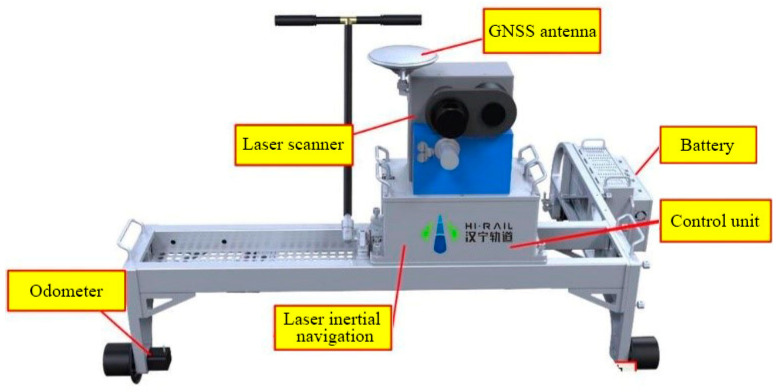
Tunnel inspection trolley (TIT) equipped with a 3D laser scanner.

In the devised system, the trolley was developed by Wuhan HiRail Transportation Technology Co., Ltd., as shown in Figure 1 [23]. The 3D laser scanner, which is PROFILER 9012 from Z + F company, is mounted on the trolley. After post-processing, the visible spectrum information (RGB) in an image can be given to the point cloud, and the shape of the tunnel internal lining can be highly restored for later point cloud modeling work. The TIT collects point cloud data at a speed of 2–5 km/h. Note that during field testing, the data collection process involves operating the inspection trolley with two personnel onboard while other team members follow behind, which requires the trolley to maintain a moderate speed to ensure safety and coordination among the team members.

### 2.3. Data Processing

By operating the TIT, a large amount of point cloud data can be collected, and the 3D model of a tunnel structure can be formed. The point cloud data are then processed to extract information on segment diameter and potential surface defects, including water leakage, segment dislocation, and spalling. The data processing steps are as follows:

Step 1: Extraction of the tunnel segment based on a point cloud using the ellipse fitting method [24].

Step 2: Point cloud projection. The following description provides a step-by-step explanation of the “Point Cloud Projection” process, involving the input of point cloud data and the fitted center position, determining the projection plane, computing projection vectors, performing point cloud unfolding, interpolation, and obtaining the output of two-dimensional coordinates and grayscale values on the projection plane for the point cloud data.

(1) Input: Point cloud data on the tunnel cross-section and the fitted center position of the cross-section.

(2) Determine Projection Plane: Based on the fitted center position of the cross-section, determine a projection plane that is perpendicular to the tunnel cross-section and passes through the center position.

(3) Compute Projection Vectors: For each point cloud data point, calculate its vector perpendicular to the projection plane, i.e., the vector from the point cloud data point to the projection plane.

(4) Point Cloud Unfolding: Project each point cloud data point onto the projection plane based on the projection vectors, resulting in the unfolded two-dimensional coordinates on the projection plane.

(5) Interpolation: For adjacent frames of point cloud data, calculate the intermediate values between them based on their corresponding grayscale values. Insert these intermediate values into the unfolded two-dimensional coordinates to complete the forward projection.

(6) Output: Obtain the two-dimensional coordinates on the projection plane and their corresponding grayscale values for the point cloud data.

Step 3: Image graying: the original cloud projection RGB is weighted to calculate the average value. The weighted calculation formula is described in Equation (1), and the weighted average value is taken as the grayscale result.

(1) $$f\left(x,\;y\right)=0.3R(x,\;y)+0.59G(x,\;y)+0.11B(x,\;y)$$

where *R*, *G,* and *B* are the three primary color channel parameters of the image, and the collective value depends on the image type.

**Figure 2:**
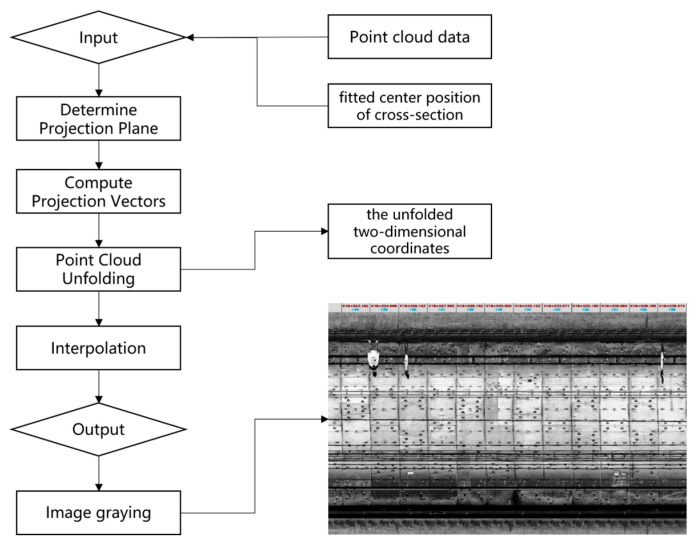
Workflow of point cloud projection and image graying.

**Figure 3:**
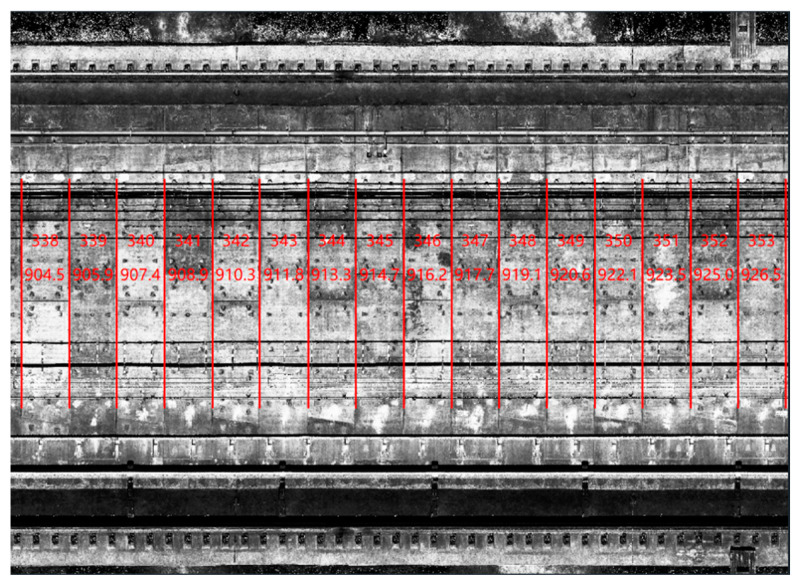
Ring locating using grayscale image processing.

With the above process, the gray-scale image of the tunnel section can be obtained, as shown in Figure 2, which clearly displays the seams of the tunnel rings. Subsequently, the seams of the tunnel rings are identified by calculating the horizontal gradient of the grayscale image [23]. Finally, the tunnel rings are identified, numbered, and located based on their seams. The result is illustrated in Figure 3, where the red regions represent the identified seams of the tunnel rings, and the number of rings is indicated on each ring.

### 2.4. Defect Identification

**Figure 4:**
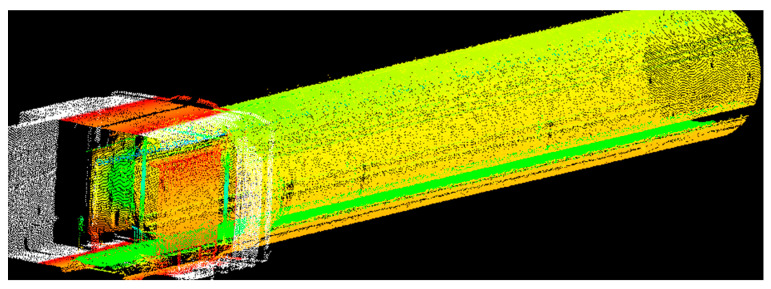
Partial scanned point cloud of Section 1.

This study completed the detection of three subway tunnel sections by operating the TIT. The lengths of the respective sections are 1.6 km (Section 1), 1.6 km (Section 2), and 2.0 km (Section 3). The construction methods, operational status, and geological and hydrogeological conditions of the three sections are presented in Table 1. With the data processing algorithm, the data on ring segment diameter, segment dislocation, water leakage, and spalling defects can be obtained. Figure 4 shows a partial point cloud scan image of Section 1, where different colors represent different tunnel structures.

#### 2.4.1. Cross-Section Deformation

**Figure 5:**
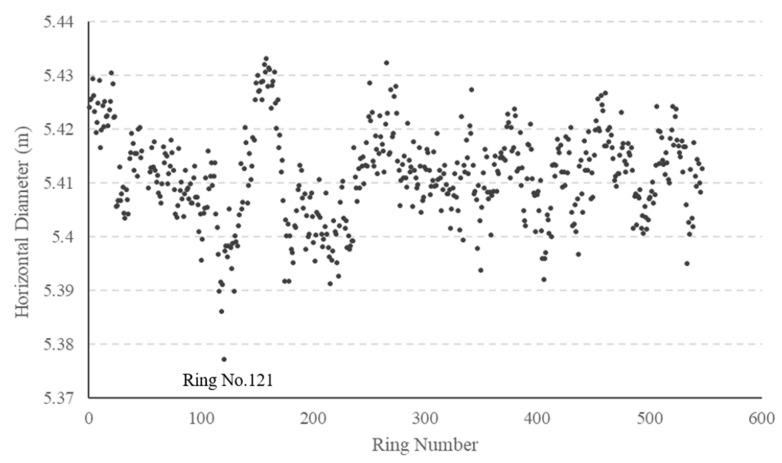
Measurement results of cross-section deformation.

The cross-section deformation data comprise the diameter, deflection angle, long axis, and short axis of each ring in the shield tunnel. Analyzing the ring diameter enables us to assess the degree of deformation in the tunnel. A total of 542 sets of effective ring data are collected in Section 1, and the horizontal diameter of each ring is presented in Figure 5.

**Figure 6:**
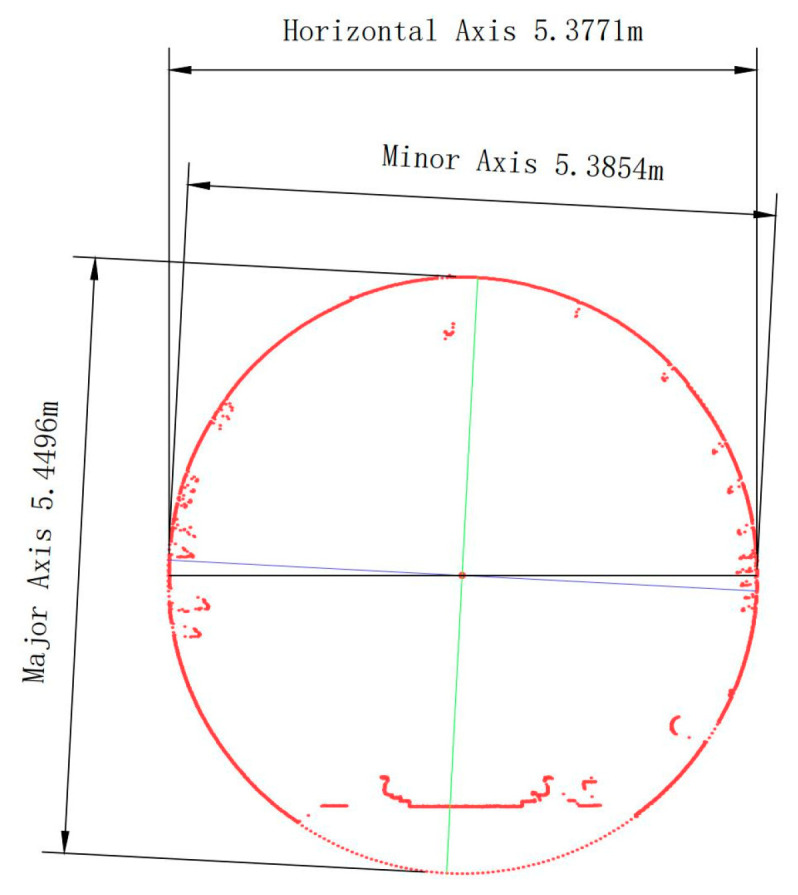
Cross-section image at ring No. 121.

In the test section, the design diameter of the rings is 5.4 m, while the average diameter measures 5.41 m. The difference between the measured horizontal diameter and the design diameter indicates the extent of cross-section deformation. It can be observed from Figure 5 that there are 8 rings with a difference of at least 3 cm, 74 rings with a difference between 2 cm and 3 cm, and 227 rings with a difference between 1 cm and 2 cm. The remaining 233 rings exhibit deformation values smaller than 1 cm. Although no severely deformed rings are found, all rings display slight deformation. Considering that tunnel deformation is widespread but not severe, it can be inferred that tunnel deformation results from natural changes occurring over time during long-term operation, unaffected by any defect factors. Figure 6 presents the cross-section shape of a typical ring (No. 121) exhibiting a significant difference between the measured and designed diameter.

#### 2.4.2. Surface Defect Detection

Given the limitations of data transmission and reception rates in the confined spaces of subway tunnels, as well as the restricted working time allowed for inspections, the selection or optimization of the detection model should meet high requirements in terms of detection accuracy and efficiency. The chosen model should also be easily deployable on lightweight and portable edge devices. Additionally, using field experiments and surveys, it is observed that surface defects in subway tunnel linings often occur around pipe joints, circumferential joints, bolt holes, and grouting holes. These defects exhibit certain spatial characteristics. Furthermore, the layout of bolt holes, cables, and pipes on the tunnel lining surfaces follows certain spatial patterns.

**Figure 7:**
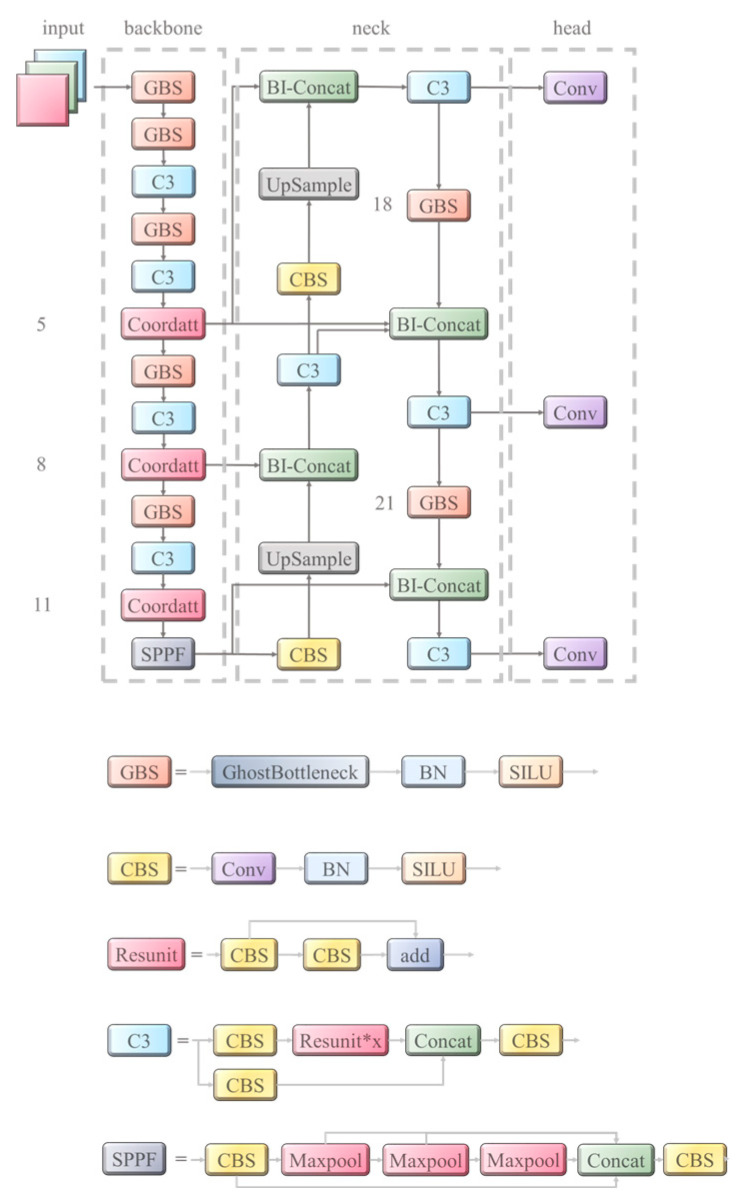
The model structure.

Based on these observations, we select YOLOv5 as the base model framework due to its high overall performance in terms of accuracy, speed, and scalability. Considering the spatial characteristics of defect occurrence, we introduce coordinate attention to help the network model quickly and efficiently focus on regions with a high frequency of defects. Furthermore, to enhance the utilization of shallow feature maps that contain more positional and structural information about the defects, we introduce a bidirectional feature pyramid network (BiFPN) in the feature fusion part of the model [25]. Lastly, ghost convolutional balancing is utilized for model parameter computation. The model structure used in this study is illustrated in Figure 7.

For the surface defect detection task, we collect original images with a resolution of 6359 × 5883 pixels. Each image corresponds to a lining segment with 16–20 rings, and the scale ratio in the actual tunnel is 200 pixels per meter. To ensure the effectiveness of the dataset annotations, automatic segmentation is performed on the original images along the ring segments and lining textures. An index is established between the segmented and original images to preserve the mileage and positional information. After segmentation, there are 872 lining images, and each segmented image corresponds to an actual lining segment with a lateral size of 1.5 m to 1.7 m and a longitudinal size of 2 m to 5 m, forming the basis for the experimental dataset. We label the images using labeling software, classifying them into four categories: ancillary facilities, bolt holes, joints, and surface defects. Additionally, considering that the collected data output type is orthophoto, we expand the data set to 4210 pieces by randomly cropping images, flipping them, adjusting their brightness and contrast, and simulating different lighting conditions in the tunnel environment. The dataset is divided into training, validation, and testing sets in an 8:1:1 ratio. The sample size of the target effective annotation box in the training set is 29,745, the sample size of the target effective annotation box in the validation set is 3647, and the sample size of the target effective annotation box in the testing set is 3584. The final experimental data division and category statistics are shown in Table 2.

This study assesses the accuracy of the detection model using precision, recall, and *F*1 score as evaluation metrics. Precision measures the ratio of correctly identified targets within a specific category, while recall indicates the ratio of accurately predicted instances of that category among the actual labels. The *F*1 score provides a comprehensive evaluation by considering both precision and recall. The calculation formulas are shown in Equations (2)–(4).

(2) $$Precision\;rate=\frac{TP}{TP+FP}$$

(3) $$Recall\;rate=\frac{TP}{TP+FN}$$

(4) $$F1\;Score=\frac{2*Precision*Recall}{\left(Precision+Recall\right)}$$

where *TP* represents the true positives, indicating the number of samples correctly detected for each category. *FP* stands for false positives, denoting the number of samples that were incorrectly classified as a particular category (detecting a target where none exists). *FN* represents false negatives, which indicate the number of samples where the model failed to detect the true targets.

**Figure 8:**
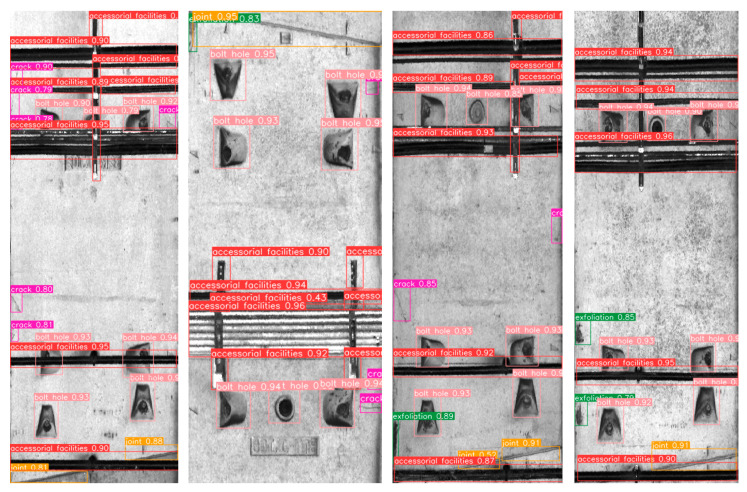
Example of detection results.

The confusion matrix presented in Table 3 provides a comprehensive evaluation of the performance of our object detection model on the test set:

The test results, displayed in Table 4, demonstrate that the model exhibits commendable detection performance for facilities and bolt-hole targets in subway tunnels. Moreover, the *F*1 scores for joints and surface defects are 0.758 and 0.714, respectively. Overall, the model successfully accomplishes the detection task with comprehensive and accurate performance. An example of the detection results is illustrated in Figure 8.

#### 2.4.3. Test Result

**Figure 9:**
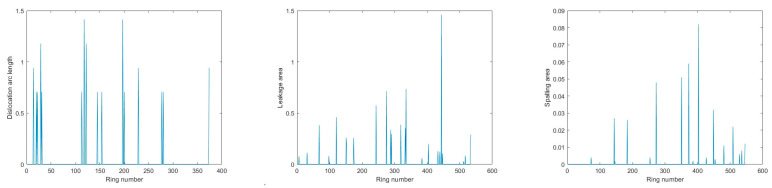
Defects identification results for Section 1.

**Figure 10:**
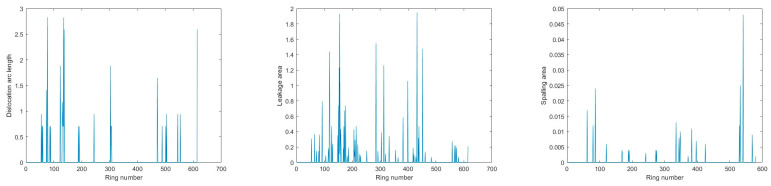
Defects identification results for Section 2.

**Figure 11:**
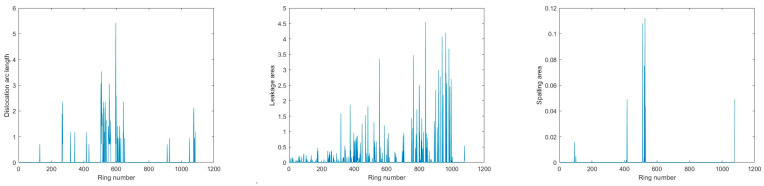
Defects identification results for Section 3.

The distribution of segment dislocation, water leakage, and spalling defects in the three sections is depicted in Figure 9, Figure 10 and Figure 11. Furthermore, typical defects in Section 1 are presented in detail as examples in this section.

(1) Segment dislocation

**Figure 12:**
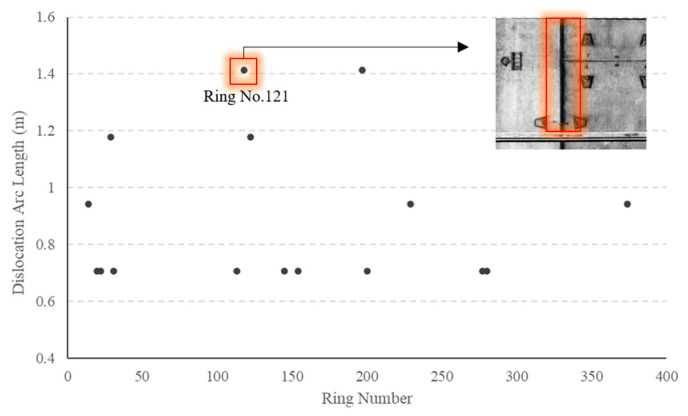
Detection results for segment dislocation.

**Figure 13:**
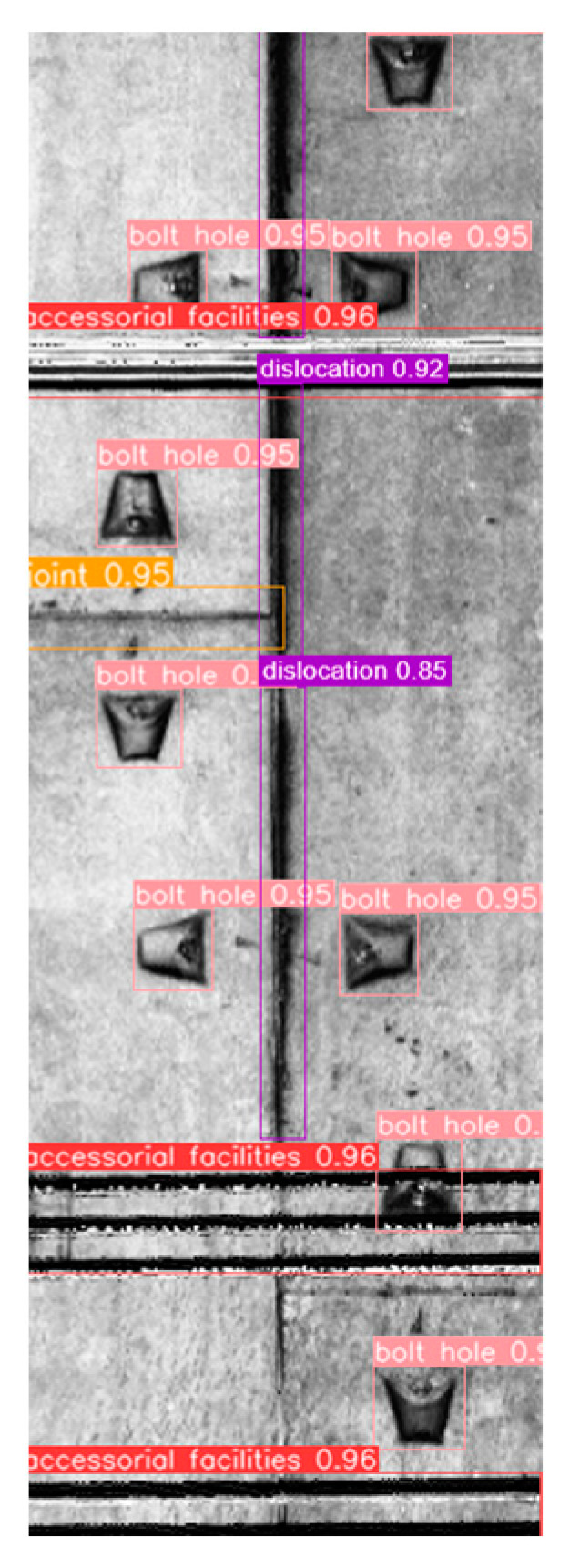
The deep learning model results at ring No. 121.

By analyzing the arc length of dislocation between ring segments, one can determine the severity of the dislocation present. A total of 16 rings with dislocation defects are discovered in Section 1, and the corresponding arc length of dislocation for each ring is displayed in Figure 12. Notably, during the examination of cross-section deformation, a significant convergence value is observed at ring No. 121 within the highlighted region. According to the definition of dislocation defect (i.e., displacement and dislocation between rings), it is probable that dislocation may occur at the adjacent areas of ring No. 121. The test result depicted in Figure 12 confirms the hypothesis by revealing the presence of dislocation defects between rings No. 118|119 and No. 122|123. Thus, it can be inferred that the deformation of the ring has a certain influence on the dislocation of the surrounding ring. Figure 13 presents the detection results of our model specifically at ring No. 121.

(2) Water leakage

**Figure 14:**
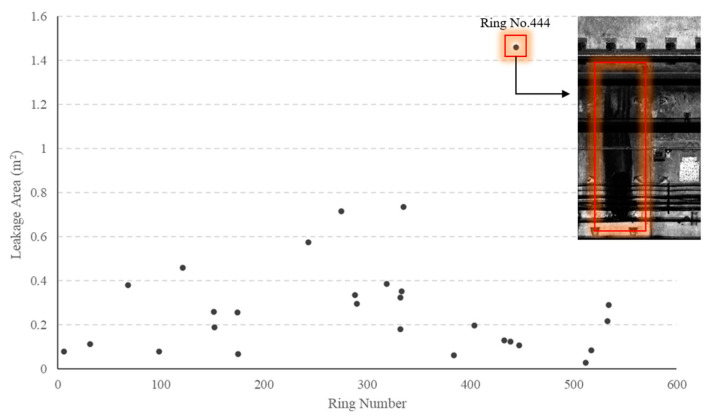
Detection results for water leakage.

**Figure 15:**
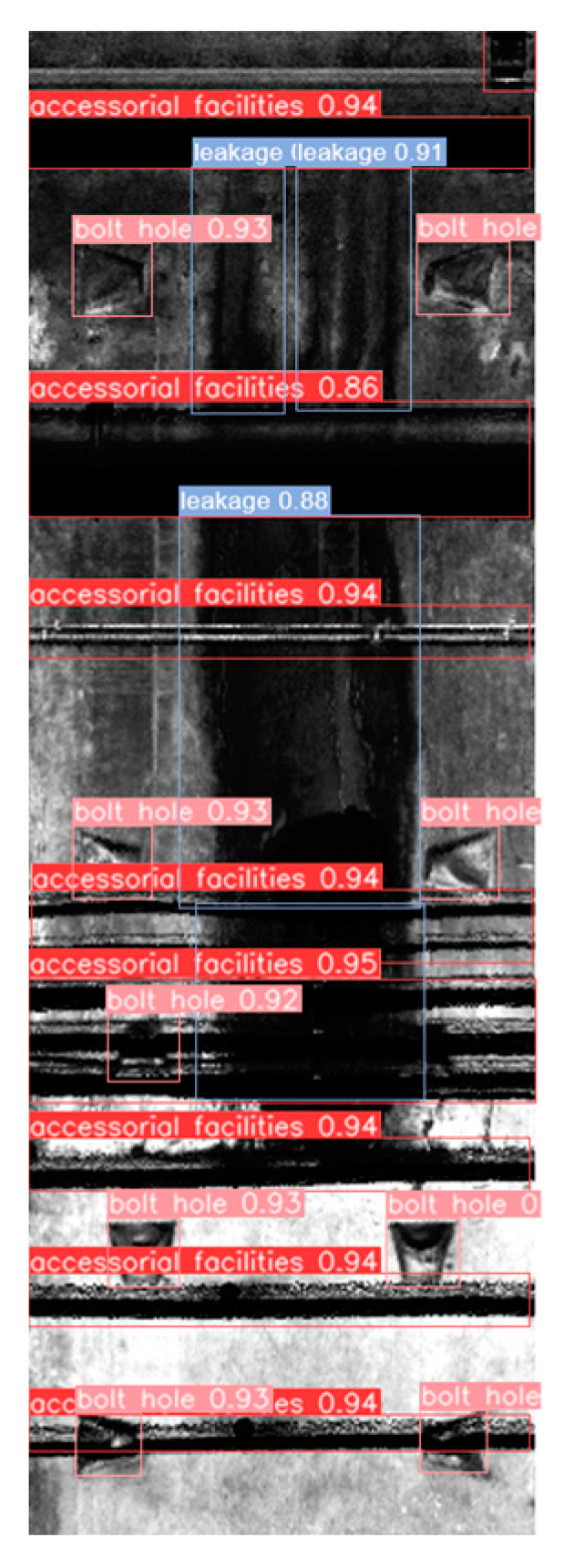
The deep learning model results at ring No. 444.

A total of 28 leakage areas are identified in Section 1, as depicted in Figure 14. The average area of water leakage within this section is 0.30 m^2^. Notably, Figure 14 highlights a significant water leakage area at ring No. 444. Further investigation of the grayscale map revealed that the water leakage position is situated at the joint between ring pieces, with no other defect detected. Therefore, it can be inferred that the loss of waterproof performance at this location is attributed to damage resulting from the splicing of ring pieces. Figure 15 displays the detection results of our model at ring No. 444.

(3) Spalling defects

**Figure 16:**
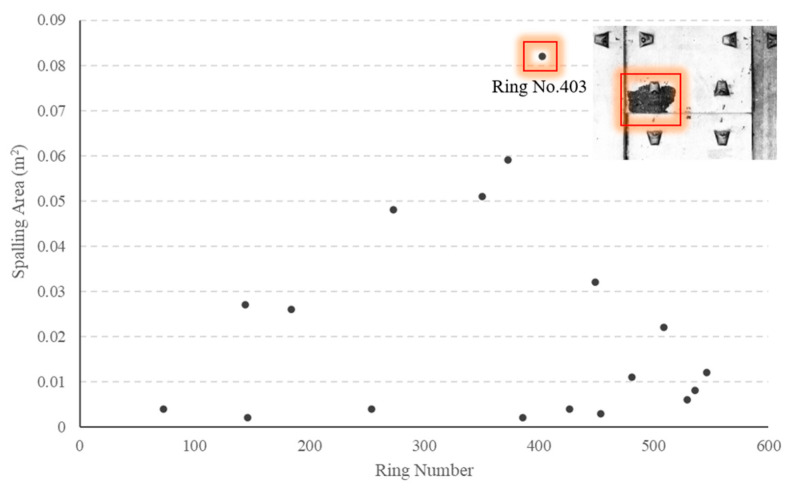
Detection results for spalling defects.

**Figure 17:**
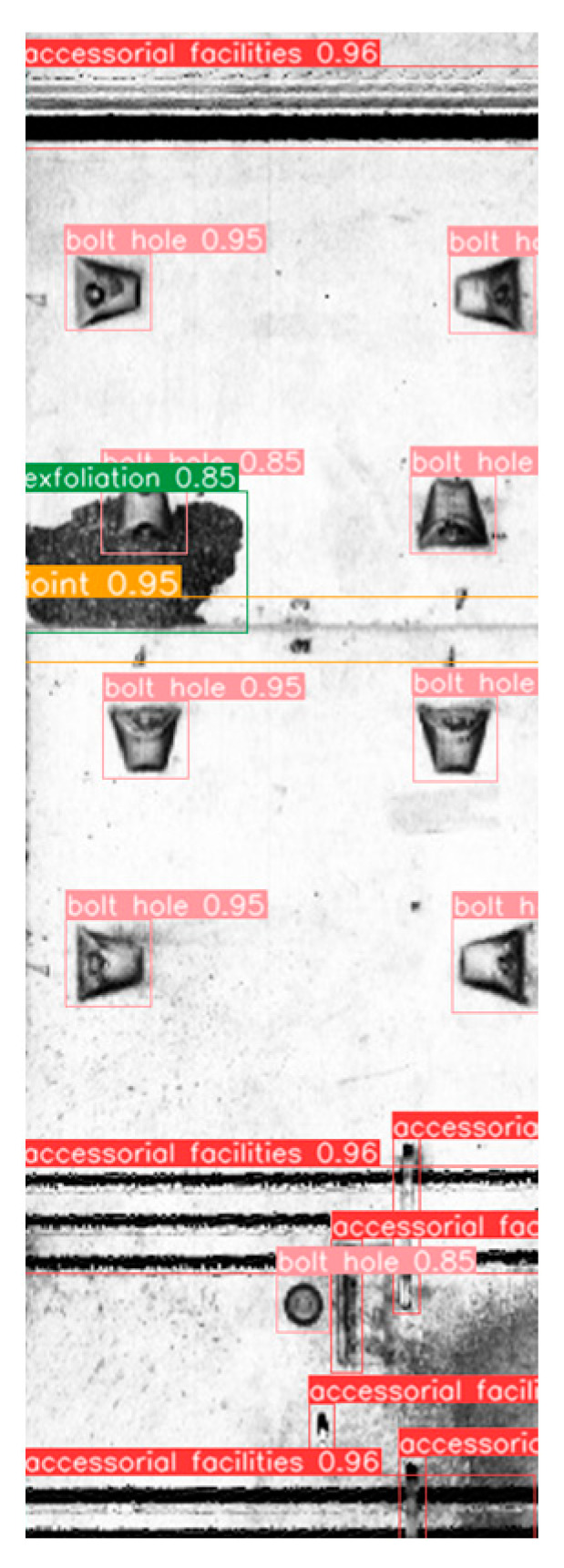
The deep learning model results at ring No. 403.

A total of 18 spalling defects are identified, as illustrated in Figure 16. The average area of spalling within the study section measures approximately 0.02 m^2^, with the largest area exceeding 0.08 m^2^ (highlighted region). The detection result of our model at ring No. 444 can be seen in Figure 17.

## 3. Defect Association Analysis

For the purpose of revealing the root cause of the surface defects, the Apriori algorithm is used to mine the association between different surface defects on the shield tunnel.

### 3.1. Introduction to the Apriori Algorithm

The Apriori algorithm is used to identify frequent item sets and establish association rules by calculating the minimum support number [26]. The advantage of this algorithm is that it can find and determine frequent itemsets and can also determine the association rules between transactions using calculation. Frequent itemsets refer to the itemsets whose occurrence times are greater than the minimum support number, while confidence refers to the probability of the occurrence of two transactions at the same time under the association rules. The frequent itemsets and association rules can be quantified using support and confidence. The main purpose of the algorithm is to mine the association rules between transactions by finding multiple frequent itemsets and verifying them using successive calculations.

#### 3.1.1. Support Count

Support count refers to the number of specific itemsets in a transaction. The value of the support count can determine the frequent transaction with the number of transactions in the itemset. Assuming itemset *I* = {*i*_1_, *i*_2_, *…*, *i_m_*} and transaction set *T* = {*t*_1_, *t*_2_, *…*, *t_n_*}, the support count of itemset *I* can be defined as:

(5) $$\sigma \left(X\right)=\left|\left\{t_{i}|X\subseteq t_{i},t_{i}\in t\right\}\right|$$

#### 3.1.2. Association Rules and Calculation Process

Association rules [27] reflect the association and dependency between multiple transactions. If several transactions have a certain association relationship, the Apriori algorithm can be used to find the frequent itemset, determine the association rules between transactions, and predict the occurrence probability of transactions. Association rules can be written in the form of *X* → *Y*. The frequent itemsets and association rules are then defined by support and confidence:

Support: Determines the minimum number of frequent itemsets.

(6) $$s\left(X\to Y\right)=\frac{\sigma \left(X\cup Y\right)}{N}$$

Confidence: Determines occurrence frequency of *Y* in transactions containing *X*.

(7) $$c\left(X\to Y\right)=\frac{\sigma \left(X\cup Y\right)}{\sigma \left(X\right)}$$

**Figure 18:**
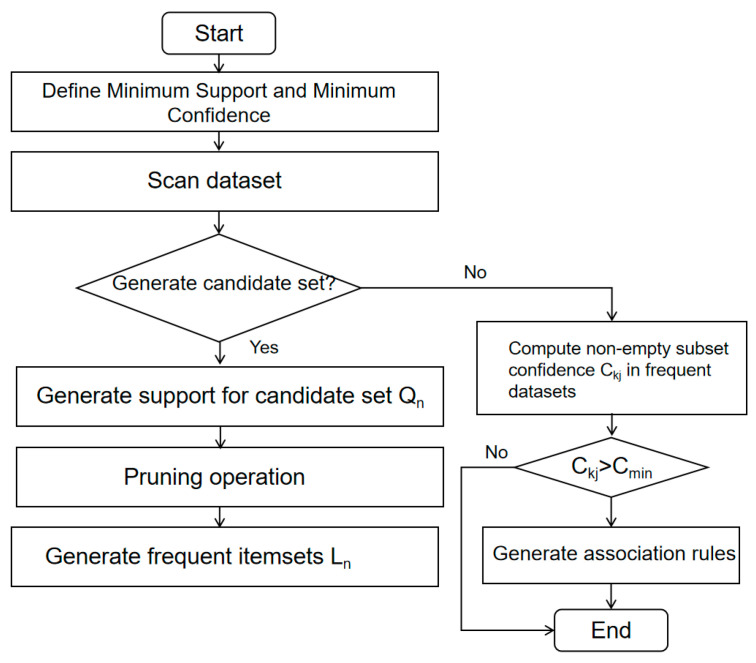
Apriori algorithm calculation process.

A strong association rule should have both high confidence and support. Assuming that the minimum confidence and support thresholds of the information dataset *D* are *C_min_* and *S_min_*, the calculation process is described as follows, and a flowchart outlining the process is shown in Figure 18.

Step 1: Search the information data set *D* to generate *L*_1_ (frequent itemset): Conduct a comprehensive search of the information dataset and count the support for each item. Items whose support count is no less than the product of the total number of transaction items in the information dataset *D* and the preset minimum support threshold are merged into *L*_1_.

Step 2: Connect *L*_k-1_ and generate a candidate *Q_k_*.

Step 3: Pruning: *L_k_* and infrequent itemsets are included in *Q_k_*. First, based on the fact that all subsets of frequent itemsets are also frequent itemsets, delete the subsets in *Q_k_* that do not meet this requirement. Then, search for the information dataset *D* to calculate the support count for the remaining itemsets in *C_k_* after the pruning operation step. Finally, find all *L_k_* larger than the predetermined minimum support threshold *S_min_*.

Step 4: Repeat the operations in Step 2 and Step 3 until *L_k_* or *Q_k_* becomes an empty set.

Step 5: Generate an association rule: Calculate the confidence value *C_kj_* for all non-empty subsets *L_kj_* in each *L_k_* separately. *C_kj_* > *C_min_* is defined as a strong association.

### 3.2. Defect Association Calculation

For the defect association calculation, the data collections are divided into several groups, and each group contains the inspection data on five rings. The minimum support number is set to three. Then, the frequent *k*-itemsets (*k* = 1, 2, 3) are determined, and their supports are calculated. Finally, the Apriori algorithm is used to establish association rules for multiple defects and calculate their confidences.

#### 3.2.1. Calculation of Support Degree of Defects

With the association calculation process, all groups with different combinations of surface defects as well as the support degree for each defect are obtained, as listed in Table 5.

#### 3.2.2. Frequent Itemset Determination

By setting the minimum support number to three, the calculation results for frequent *k*-itemsets (*k* = one, two, three) are obtained, as listed in Table 6.

#### 3.2.3. Confidence Calculation

(1) Determine the association rules: nine kinds of association rules are set in this calculation, which are “dislocation, spalling → water leakage”, “water leakage, spalling → dislocation”, “dislocation, water leakage → spalling”, “water leakage → spalling”, “water leakage → dislocation”, “dislocation → water leakage”, “dislocation → spalling”, “spalling → water leakage”, and “spalling → dislocation”.

(2) Derive the confidence of “dislocation, spalling → water leakage”, “water leakage, spalling → dislocation”, “dislocation, water leakage → spalling” using the frequent two and three itemsets. Note that the left side of “→” is the existing defect and the right side denotes the possible simultaneous defect. Confidence refers to the probability that when one defect exists, another defect exists at the same time. The confidence is defined as the number of occurrences of the frequent three-itemset divided by the number of occurrences of the corresponding frequent two-itemset.

(3) Similarly, the confidence of the other six association rules can be derived from the frequent two-itemset and the frequent one-itemset.

With the above calculation process, the results for confidence are summarized in Table 7:

### 3.3. Discussion

Based on the above calculation of the association rules for the multiple defects, it is found that:

In different sections, the confidence level for each association is different, for example, the confidence level of “Dislocation, Spalling → Water leakage” and “Spalling → Water leakage” in Section 3 is 100%, while the confidence level of the other two sections is less than 40%. There are also commonalities in the differences, such as the confidence of “Water leakage → Spalling” in all three sections is less than 20%.

(1) On average, when there is a water leakage defect in the tunnel, within the range of five rings around the defect, the probability of a dislocation defect is 23.28%, and the probability of a spalling defect is 13.00%. When there is a dislocation defect in the tunnel, within the range of five rings around the defect, the probability of a spalling defect is 33.77%, and the probability of a water leakage defect is 53.61%. When there is a spalling defect in the tunnel, within the range of five rings of the defect, the probability of a water leakage defect is 51.01%, and the probability of a dislocation defect is 43.73%.

(2) When there are both dislocation and spalling defects at the same location, the probability of a water leakage defect is 57.78% within the range of five rings. When there are both water leakage and spalling defects at the same location, the probability of a dislocation defect is 55.56% within the range of five rings. When there are dislocation and water leakage defects at the same location, the probability of a spalling defect is 31.88% within the range of five rings.

The results show that the confidence levels for some association rules are low, with the lowest “Water leakage → Spalling” having a confidence of only 13.00%. For such association rules with low confidence, taking this as an example, it can be considered that the probability of spalling due to water leakage is not high. In comparison, some association rules have quite high confidence levels, such as “dislocation, spalling → water leakage”, which has a confidence of 57.78%. Therefore, in the tunnel defect detection work, when dislocation and spalling defects are found, it is necessary to detect a water leakage defect around the dislocation and spalling defects. It can be inferred from the causes and phenomena of the three defects that the structural damage in the tunnel ring is caused by segment dislocation and spalling, which affects the waterproof performance and thus leads to the occurrence of water leakage defects.

It is worth noting that the Apriori algorithm focuses on identifying associations based on itemsets and support values, while parameters such as density, spatial distribution, and interactions between different types of defects are also important factors in analyzing tunnel behavior. They may require different methodologies or techniques to be investigated comprehensively. Therefore, future research can explore algorithms that incorporate a more detailed analysis of certain parameters. By comparing these algorithms with the approach presented in this paper, we can gain a more comprehensive understanding of the relationships between defects in the tunnel. Incorporating comparative analyses would provide a more comprehensive evaluation of different approaches and enhance the robustness of our findings.

## 4. Conclusions

In recent years, the issue of surface defects on shield tunnels has garnered increasing attention from engineers and researchers due to the rapid development of subways. The primary focus is on ensuring safe operation and enhancing the serviceability of the tunnel structures, making the efficiency and accuracy of defect detection paramount. This paper provides a comprehensive overview of common shield tunnel defects, including segment dislocation, water leakage, spalling, and cross-section deformation. In terms of detection technology, leveraging the unique advantages of laser scanning technology in subway inspection, this study uses a laser scanning-based system for in situ tunnel inspection. With the inspection trolley, a vast amount of point cloud data on a line section is acquired. By using image processing algorithms, pertinent information regarding the tunnel, including segment dislocation, water leakage, and spalling, can be extracted from the grayscale images.

Using the defect dataset as a basis, a defect association analysis is conducted using the Apriori algorithm. This analysis aims to uncover the underlying causes of surface defects. A total of nine sets of association rules are set up, and their respective confidence levels are calculated. Subsequently, the interrelationship among different defects is examined. The findings indicate that the two types of defects with the highest correlation degree are “dislocation → water leakage” followed by “spalling → water leakage”, and the lowest correlation degree (i.e., “water leakage → spalling”) has a confidence of 13.00%. Significantly, some correlations between various defects in the tunnel are quite strong. Among them, the confidence of the association rule “dislocation, spalling → water leakage” is as high as 57.78%. Thus, when detecting dislocation and spalling defects, it becomes crucial to prioritize the identification of potential water leakage issues in the vicinity.

The results of the association analysis have practical implications for preventing subway tunnel defects by enabling the prediction of additional types of defects once one is detected. Taking the association rule “dislocation → water leakage” as an example, when segment dislocation is detected in the tunnel, there is a 53.61% probability that there will be water leakage in the surrounding areas. Consequently, it is essential to promptly develop and implement water leakage treatment measures to prevent or address potential leaks. Throughout the operation of subway tunnels, heightened attention should be given to the interconnectedness among different structural defects, as this can significantly enhance both subway operation safety and infrastructure reliability.

## Figures and Tables

**Table 1:** Detailed information on the three sections.

	Construction Method	Operational Status	Geological Conditions	Hydrogeological Conditions
Section 1	Shield tunneling	In operation	The original landform is an alluvial plain with predominant residual cohesive soil and highly weathered tuffaceous sandstone within the tunnel area. The surrounding rock has poor stability.	Surface water is not developed, and the groundwater level mainly exists in the gravel and sand layers. The formation has high permeability and good water-bearing properties.
Section 2	Shield tunneling	In operation	The original landform consists mainly of alluvial plains and some plateaus. The geological layers within the tunnel area consist of silty clay, clay, and gravel and sand layers.	Surface water is not developed, and the groundwater level mainly exists in the gravel and sand layers. The formation has high permeability and good water-bearing properties.
Section 3	Shield tunneling	Under construction	The original landform includes plateaus and hills with significant topographical variations. Within the fault zone, there are moderately weathered fragmented rocks.	Surface water primarily consists of rivers and a few channels. There is hydraulic connection between the river water and the confined aquifers in the quaternary loose rock formations.

**Table 2:** Division of experimental dataset.

Dataset	Sample Count	Facility Count	Bolt Hole Count	Joint Count	Surface Defect Count
Training set	3368	13,491	9492	4798	1964
Validation set	421	1685	1143	575	244
Testing set	421	1740	1077	543	224
Total	4210	16,916	11,712	5916	2432

**Table 3:** Confusion matrix.

	Predicted Facility	Predicted Bolt Hole	Predicted Joint	Predicted Surface Defects	Missed Detection
Actual Facility	1547	2	16	5	170
Actual Bolt hole	1	1039	0	7	30
Actual Joint	84	1	365	14	89
Actual Surface defects	13	27	35	142	7
Absence of Targets	2	1	4	5	/

**Table 4:** Testing results.

Evaluation Metrics	Facilities	Bolt Holes	Joints	Surface Defects	Average
**Precision**	0.949	0.971	0.869	0.821	0.903
**Recall**	0.889	0.965	0.672	0.632	0.789
**F1**	0.918	0.968	0.758	0.714	0.842

**Table 5:** Calculation results for defect supports.

Defect Type	Support Degree S1	Support Degree S2	Support Degree S3
Dislocation	33.33%	30.30%	23.53%
Water leakage	53.85%	69.70%	91.60%
Spalling	46.15%	33.33%	6.72%
Dislocation, Water leakage	20.51%	10.61%	15.13%
Dislocation, Spalling	12.82%	12.12%	4.20%
Water leakage, Spalling	7.69%	13.64%	6.72%
Dislocation, Water leakage, Spalling	5.13%	4.54%	4.20%

**Table 6:** Calculation results for frequent *k*-itemsets (*k* = one, two, three).

Frequent One Term Set	Section 1	Section 2	Section 3
Dislocation	13	20	28
Water leakage	21	46	109
Spalling	18	22	8
**Frequent binomial set**	**Section 1**	**Section 2**	**Section 3**
Dislocation, Water leakage	8	7	18
Water leakage, Spalling	3	9	8
Dislocation, palling	5	8	5
**Frequent trinomial set**	**Section 1**	**Section 2**	**Section 3**
Dislocation, Water leakage, Spalling	2	3	5

**Table 7:** Calculation results for confidence.

Defect Type	Confidence S1	Confidence S2	Confidence S3	Average
Water leakage → Dislocation	38.10%	15.22%	16.51%	23.28%
Water leakage → Spalling	14.28%	17.39%	7.34%	13.00%
Dislocation → Spalling	38.46%	45.00%	17.86%	33.77%
Dislocation → Water leakage	61.54%	35.00%	64.29%	53.61%
Spalling → Dislocation	27.78%	40.91%	62.50%	43.73%
Spalling → Water leakage	16.67%	36.36%	100%	51.01%
Dislocation, Spalling → Water leakage	40.00%	33.33%	100%	57.78%
Water leakage, Spalling → Dislocation	66.67%	37.50%	62.50%	55.56%
Dislocation, Water leakage → Spalling	25.00%	42.86%	27.78%	31.88%

## Data Availability

All data, models, and codes that support the findings of this study are available from the corresponding author upon reasonable request.
